# The relation between media promotions and service volume for a statewide tobacco quitline and a web-based cessation program

**DOI:** 10.1186/1471-2458-11-939

**Published:** 2011-12-16

**Authors:** Barbara A Schillo, Andrea Mowery, Lija O Greenseid, Michael G Luxenberg, Andrew Zieffler, Matthew Christenson, Raymond G Boyle

**Affiliations:** 1 ClearWay Minnesota SM, 8011 34th Avenue South, Minneapolis, MN 55425, USA; 2 Professional Data Analysts, Inc., 219 Main Street SE, Suite 302, Minneapolis, MN 55414, USA; 3 University of Minnesota, 56 East River Road, Minneapolis, MN 55455, USA

## Abstract

**Background:**

This observational study assessed the relation between mass media campaigns and service volume for a statewide tobacco cessation quitline and stand-alone web-based cessation program.

**Methods:**

Multivariate regression analysis was used to identify how weekly calls to a cessation quitline and weekly registrations to a web-based cessation program are related to levels of broadcast media, media campaigns, and media types, controlling for the impact of external and earned media events.

**Results:**

There was a positive relation between weekly broadcast targeted rating points and the number of weekly calls to a cessation quitline and the number of weekly registrations to a web-based cessation program. Additionally, print secondhand smoke ads and online cessation ads were positively related to weekly quitline calls. Television and radio cessation ads and radio smoke-free law ads were positively related to web program registration levels. There was a positive relation between the number of web registrations and the number of calls to the cessation quitline, with increases in registrations to the web in 1 week corresponding to increases in calls to the quitline in the subsequent week. Web program registration levels were more highly influenced by earned media and other external events than were quitline call volumes.

**Conclusion:**

Overall, broadcast advertising had a greater impact on registrations for the web program than calls to the quitline. Furthermore, registrations for the web program influenced calls to the quitline. These two findings suggest the evolving roles of web-based cessation programs and Internet-use practices should be considered when creating cessation programs and media campaigns to promote them. Additionally, because different types of media and campaigns were positively associated with calls to the quitline and web registrations, developing mass media campaigns that offer a variety of messages and communicate through different types of media to motivate tobacco users to seek services appears important to reach tobacco users. Further research is needed to better understand the complexities and opportunities involved in simultaneous promotion of quitline and web-based cessation services.

## Background

Telephone-based tobacco cessation counseling is now widely available [1], with considerable evidence supporting the effectiveness of this population-based approach in helping individuals quit using tobacco [2-4]. Recently, web-based cessation programs have become increasingly available. While the literature supporting web-based intervention is more limited than that of telephone counseling, a growing body of evidence supporting the effectiveness of Internet cessation interventions is emerging [5-7]. The web by its nature has the potential to expand reach of evidence-based cessation interventions to tobacco users worldwide [8]. In addition, web-based technology continues to evolve to include highly interactive and tailored tools for tobacco cessation [9], which have been found to be more effective than generic approaches [10,11].

Mass media is an important tool in making tobacco users aware of cessation services and in motivating this population to use these services. Prior research has demonstrated a connection between media campaigns and calls to tobacco quitlines, showing that mass media campaigns can effectively increase calls to quitlines [12,13] and that call volume is strongly related to targeted rating point levels (TRPs) [14,15]. TRPs are a standard unit of measurement for media delivery that reflect the reach and frequency of an advertisement with reach being the total percentage of the targeted population that is exposed to the advertisement and frequency defined as the number of times individuals in the targeted population saw it on average [16]. In addition, placement of advertisements (day, time and program) and types of advertisements (event and message variation) have been shown to positively influence quitline call volume [13-15,17,18].

We are aware of no studies, however, that have examined the impact of media campaigns on the use of stand-alone web-based cessation services or the relation between mass media campaigns and multiple tobacco cessation programs. As more states and employers consider the addition of online services to their cessation offerings--either through the addition of stand-alone web programs or integrated phone and web services--more information is needed to understand how to promote these cessation options effectively and how multiple services and their promotions impact each other.

During the period of this study (July 2005 to March 2008), cessation services were offered for Minnesota smokers through the QUITPLAN^® ^telephone counseling program and the quitplan.com stand-alone web program. During this same time period, ClearWay Minnesota ^SM ^ran three statewide media campaigns: a tobacco cessation campaign; a secondhand smoke campaign; and a campaign to educate about a new statewide smoke-free law. The cessation campaign promoted the QUITPLAN quitline number and the QUITPLAN website. Each television ad for the cessation campaign included both the telephone number and the website url. Each radio and print ad included either the telephone number or the url and both types of ads were placed equally. The secondhand smoke and smoke-free law campaigns did not promote the QUITPLAN quitline number or the QUITPLAN website but were included in this study based on observations that non-cessation based campaigns impact service volumes.

**Figure 1:**
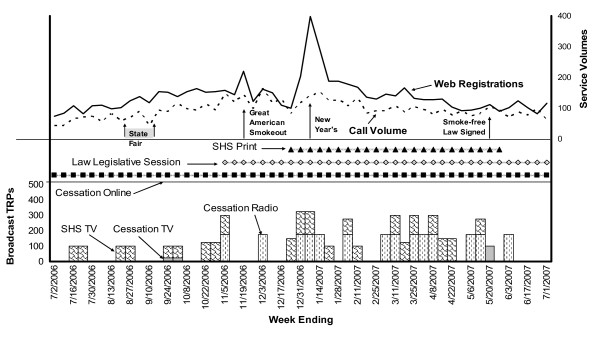
**Paid media, earned media, and service volume included in statistical models, July 2006-June 2007**.

The cessation and secondhand smoke campaigns provided a combination of "how to quit" and "why to quit" messages. Research has demonstrated that this combination is effective in motivating smokers to quit [15,17,19]. The ads included in this analysis include messaging around: 1) a better way to quit smoking; 2) health consequences of smoking and secondhand smoke; and 3) awareness of a statewide smoke-free law. An illustration of service volume (weekly calls to the cessation quitline and weekly registrations to the web-based cessation program) and of both paid and earned media for a 12-month period of the study (July 2006 to June 2007) is presented in Figure 1. (An illustration of service volume and media for the entire study period--July 2005 to March 2008--is presented in Additional file 1.)

This study addresses a gap in the literature by describing results from an analysis that explored the relation between mass media campaigns and service volumes for both a quitline and web-based cessation program to try to understand the effect of TRP levels, different media campaigns, and different media types on cessation service volumes.

## Methods

Two specific research questions were addressed through a retrospective analysis of data from July 2005 to March 2008. We used a multivariate time series design to investigate whether or not media volume during a week is predictive of quitline and web volumes during the same week that the media is run. The first question examined whether or not overall statewide broadcast mass media efforts were related to individuals seeking cessation services, after controlling for other external and earned media events that may affect an individual's interest in cessation services. To address this question, we examined the relation between the TRPs for television and radio advertisements and weekly calls to a cessation quitline and weekly registrations to a web-based cessation program. The second question examined the relative influence of the campaigns and media types on service volumes. We looked at the relation between the type of media (television, radio, print, on-line, and out-of-home [i.e., bus sides, billboards, mall advertising]), the three media campaigns that ran during the time period (cessation, secondhand smoke, and smoke-free law) and weekly calls to the quitline and weekly registrations on the web program.

Analyses for both research questions controlled for events hypothesized to promote cessation for the study period. There is evidence that smoke-free laws [20] and tax increases [21] encourage smokers to quit and that these policy shifts combined with earned media can increase consumer demand for cessation services [22]. In addition, the QUITPLAN telephone number and url are often featured as part of external and earned media events and we have observed the impact of earned media promotions on increased calls to the QUITPLAN quitline and registrations on quitplan.com. Finally, a proxy for economic stress (i.e., gas prices) was included in the model. Evidence suggests that economic stress has the potential to impact cessation [23] and during the time period of this study, both gas prices and economic stress rose as the United States economy went into recession.

### Variables

The data sources for this analysis included administrative data on advertising and promotions and call volume and web registration levels provided by cessation service vendors. The variables used in this study are presented in Table 1. The two outcome measures for the analyses were the number of weekly calls to the quitline and weekly registrations on the web program as reported by the contracted service vendors for the period of July 2005 to March 2008. Because both call volume and web registration distributions were positively skewed, a log transformation was performed on each variable prior to the analysis to normalize distributions.

**Table 1:** Variables included in the multivariate regression analyses

Variables	Description	Question One	Question Two
Dependent Variables			

Quitline call volume	Natural log of the number of weekly calls to the quitline toll-free number as recorded in telephone service call records	X	X
Web program registrations	Natural log of the number of weekly online registrations for the web cessation program	X	X

Independent Variables			

Television and radio TRPs	Sum of weekly TRPs for television and radio cessation ads, secondhand smoke ads, and smoke-free law ads	X	
Cessation TV campaign	A binary variable for each week indicating if there was television advertising for the cessation campaign		X
Cessation print campaign	A binary variable for each week indicating if there was print advertising for the cessation campaign		X
Cessation radio campaign	A binary variable for each week indicating if there was radio advertising for the cessation campaign		X
Cessation online campaign	A binary variable for each week indicating if there was online advertising for the cessation campaign		X
Cessation out-of-home campaign	A binary variable for each week indicating if there was out-of-home advertising for the cessation campaign		X
Cessation other campaign	A binary variable for each week indicating if there was other advertising for the cessation campaign		X
Secondhand smoke TV campaign	A binary variable for each week indicating if there was TV advertising for the secondhand smoke campaign		X
Secondhand smoke print campaign	A binary variable for each week indicating if there was print advertising for the secondhand smoke campaign		X
Secondhand smoke radio campaign	A binary variable for each week indicating if there was radio advertising for the secondhand smoke campaign		X
Secondhand smoke online campaign	A binary variable for each week indicating if there was online advertising for the secondhand smoke campaign		X
Secondhand smoke out-of-home campaign	A binary variable for each week indicating if there was out-of-home advertising for the secondhand smoke campaign		X
Secondhand smoke other campaign	A binary variable for each week indicating if there was other advertising for the secondhand smoke campaign		X
Smoke-free law TV campaign	A binary variable for each week indicating if there was TV advertising for the smoke-free law campaign		X
Smoke-free law restroom campaign	A binary variable for each week indicating if there was restroom advertising for the smoke-free law campaign		X
Smoke-free law radio campaign	A binary variable for each week indicating if there was radio advertising for the smoke-free law campaign		X

Covariates			

Week	Week number; included to account for the linear trend in the data	X	X
Previous week's call volume	A one week lag of the natural log of number of calls; included to account for week-to-week correlation in the residuals	X	X
Previous week's web registrations	A one week lag of the natural log of number of web registrants; included to account for week-to-week correlation in the residuals	X	X
Gas Price	Four-week moving average of gas price; included as a measure of economic stress	X	X
Health Impact Fee	75 cents increase in the cost of cigarettes (August 1, 2005); a binary variable coded 1 the week of 7/31/2005 (Peter Jenning's death coincides with Health Impact Fee)	X	X
Quit To Live	ABC News month long special Quitting to Live: Fighting Lung Cancer - promotes 1-800-QUIT-NOW; a binary variable coded 1 the weeks of 10/30/2005 through 11/27/2005	X	X
Big Butt Billboard	Big Butt spectacular billboard launches; a binary variable coded 1 the weeks of 11/27/2005 through 12/25/2005	X	X
Freedom To Breathe legislative session	Freedom to Breathe (statewide smoke free law) legislation session debates (Nov. 2006 - June 2007); a binary variable coded 1 the weeks of 10/29/2006 through 6/24/2007	X	X
Freedom To Breathe Law	Freedom to Breathe signed into law (May 16, 07); a binary variable coded 1 the week of 5/13/2007	X	X
Age Progression Software	Launched age-progression software a binary variable coded 1 the week of 06/24/2007	X	X
Freedom To Breathe Implementation	Freedom to Breathe implements (October 1, 2007); a binary variable coded 1 the week of 9/30/2007	X	X
Great American Smokeout	Great American Smokeout (third Thursday of November - every year); a binary variable coded 1 the weeks of 11/13/2005, 11/12/2006, and 11/11/2007	X	X
New Year's Resolutions	New Year resolution; a binary variable coded 1 the weeks of 1/1/2006, 12/31/2006, and 12/30/2007	X	X
Minnesota State Fair Booth	QUITPLAN Services booth at the Minnesota State Fair; a binary variable coded 1 the weeks of 8/21/05-9/4/05, 8/20/06-9/3/06, and 8/19/07-9/2/07	X	X

The independent variables in the study were different for each research question. For the first question, weekly television and radio TRPs were used as the media predictor. This variable was the sum of the weekly television and radio TRPs for cessation media, the secondhand smoke campaign, and the smoke-free law campaign. For the second question, indicator variables designating on and off weeks for the television, radio, print, online, and out-of-home advertisements for each of the three media campaigns were used. We used indicator variables instead of TRPs as TRPs were not uniformly available for all media types of interest in the second analysis. We used 15 media predictors: six types of media (television, radio, online, print, out-of-home ads, and other) for both the cessation campaign and the secondhand smoke campaign, and three media types for the smoke-free law campaign (television, radio, and restroom). All data on television, radio, print, online advertising buys, and out-of-home promotional efforts were provided by the advertising agency responsible for the media campaign.

Fourteen covariates were included in each analysis. These covariates included a variable to account for a linear trend in the data, and two lagged variables to account for the week-to-week correlation in the number of calls or web registrations in 1 week with the previous week's levels. Including the previous week's volume as predictors models local history and accounts for the autocorrelation of residuals in the regression as evidenced by the improved Durbin-Watson autocorrelation statistic from 1.16 to 2.27 for call volume and from 1.40 to 2.23 for web registrations. An additional covariate included in the models was the 4-week moving average of Minnesota regular conventional retail gas prices as a proxy measure of economic stress in the model [24]. Lastly, 10 binary variables dummy coded to indicate whether earned media coverage or external events may have heightened interest in cessation during a particular week were included in the models.

### Statistical modeling

For each research question, a separate regression model was fitted. The models controlled for the impact of external and earned media events related to tobacco issues that occurred during the study period to allow for the impact of the advertising to be examined. Two multivariate regression analyses were conducted using the statistical software package, R (R Development Core Team, 2009) and IBM^® ^SPSS^® ^[18]. In this study, the two outcome variables (natural log of number of calls and natural log of number of web registrations) were moderately to highly correlated (r = 0.53). In the GLM multivariate analysis, both dependent variables were evaluated simultaneously when determining which predictors are included in the model. However, the presentation of the model coefficients shown for each dependent variable (using the multivariate GLM) were based on the univariate model not adjusted for the other dependent variable. The variance inflation factors associated with each of the predictors in the models were also examined. Each of the variance inflation factors were well under the commonly used threshold of 10 indicating that multicollinearity is likely not an issue for the model.

For each model, the standardized betas (β) were calculated. The standardized beta represents the standard deviation change in the predicted value of the dependent variable (in this case, log of call volume or web registrations) that is associated with a one standard deviation change in an independent variable. Additionally, partial eta square values were calculated for both models. Partial eta squared is the measure of effect size provided by SPSS for multivariate analyses. It is interpreted as the proportion of the total error variation explained by the factor, after partialling out (excluding) other factors from the total non-error variation. Since it is the contribution of a particular variable calculated as if it were the only variable, the total partial eta squared values for a model can sum to more than 100%.

The first regression model examined the overall relation between ClearWay Minnesota's television and radio advertisements and weekly calls to the quitline and weekly web registrations. The model was fitted to the data and included the 14 covariates along with one media predictor of weekly broadcast TRPs.

The second regression model examined the relative impact of the three media campaigns run on six types of media on weekly quitline calls and weekly web registrations. The model was fitted to the data and included the 15 media predictors (six types of media for both the cessation campaign and the secondhand smoke campaign and three media types for the smoke-free law campaign) and the 14 external and earned media covariates. Both models were revised and simplified through a series of model comparisons.

## Results

### Question one: television and radio TRPs and quitline calls and web registrations

The model found a positive relation between overall television and radio weekly broadcast TRPs and weekly calls to the quitline and weekly web registrations, after controlling for the linear trend in the data, economic stress levels and important external and earned media events that may have impacted interest in quitting (See Table 2). After back-transforming the parameter estimates from natural log units into web registrations or call volume counts, we find that one person called the quitline for every 14 weekly broadcast TRPs. We find that approximately one person registered for the web program for every seven weekly broadcast TRPs.

**Table 2:** Question one: summary of multivariate findings for relation between radio and television TRPs, weekly calls to the quitline, and web program registrations

Variable	Quitline Call Volume			Web Program Registrants		
	
	Standardized Beta	**Partial Eta**^**2**^	**Sign**.	Standardized Beta	**Partial Eta**^**2**^	**Sign**.
Week (linear trend in data)	-0.24	0.04	**	--	--	ns
Gas Price (proxy for economic stress)	-0.31	0.07	**	-0.39	0.12	***
Statewide Smoke-Free Law - Legislative Session	--	--	ns	--	--	ns
Great American Smokeout	0.14	0.03	*	0.18	0.05	**
New Year's Resolutions	--	--	ns	0.30	0.14	***
TV/Radio Weekly Broadcast TRPs	0.20	0.06	**	0.28	0.12	***

*p < 0.10; **p < 0.05; ***p < .001

Table 2 presents the standardized betas and partial eta square values associated with each significant variable for the first research question. For weekly quitline calls, while there was a positive relation between weekly broadcast TRPs and quitline calls, we find that the price of gas, a proxy for economic stress, had a stronger relation. The Great American Smokeout was also related to quitline calls but at a somewhat lower level. For weekly web program registration levels, we find that gas price had the strongest relation with predicted registration levels followed closely by New Year's resolutions and television and radio broadcast TRPs. The Great American Smokeout was also related to web registrations at a lower level.

For question one, the multivariate regression results for the final fitted model were statistically significant, *F*(6, 131) = 11.25, *p *< .001, adj. R-squared = 0.31 (Call Volume); and *F*(6, 131) = 16.76, *p *< .001, adj. R-squared = 0.41 (Web Registrations).

### Question two: media campaigns and types and quitline calls and web registrations

Multivariate findings for the relation between media types and campaigns and weekly calls to the quitline and weekly registrations to the website are presented in Table 3. Both online advertising related to the cessation campaign and the print advertising associated with the secondhand smoke campaign were positively related to weekly calls to the quitline, after controlling for the linear trend in the data and other important media events. Each additional week that the cessation campaign ran online resulted in an additional 22 callers to the quitline. Each additional week that print media associated with the secondhand smoke campaign was run, an additional 19 individuals called the quitline.

**Table 3:** Question two: summary of multivariate findings for relations between media types and campaigns, weekly calls to the quitline, and web program registrations

Variable	Quitline Call Volume			Web Program Registrations		
	
	Standardized Beta	**Partial Eta**^**2**^	**Sign**.	Standardized Beta	**Partial Eta**^**2**^	**Sign**.
Week (linear trend in data)	-0.30	0.03	**	-0.29	0.05	**
Gas Price (proxy for economic stress)	--	--	ns	-0.31	0.15	***
Previous week's call volume	0.39	0.14	***	--	--	ns
Previous week's web registration level	0.24	0.06	**	0.37	0.22	***
Health Impact Fee (75 cent per pack fee added 8/1/05)	--	--	ns	0.15	0.08	**
Great American Smokeout	--	--	ns	0.18	0.11	***
New Year's Resolutions	0.11	0.03	*	0.27	0.22	***
Minnesota State Fair Booth	--	--	ns	0.15	0.07	**
Print - Secondhand Smoke Campaign	0.20	0.04	**	--	--	ns
Radio - Secondhand Smoke Campaign	--	--	ns	-0.18	0.07	**
Radio - Smoke-free Law Campaign	--	--	ns	0.23	0.15	***
Radio - Cessation Campaign	--	--	ns	0.09	0.02	*
TV - Cessation Campaign	--	--	ns	0.33	0.25	***
Online - Cessation Campaign	0.30	0.03	**	--	--	ns

*p < 0.10; **p < 0.05; ***p < .001

For weekly web registrations, after controlling for the linear trend and important events, we find that television and radio broadcasts of the cessation campaign and radio broadcasts of the smoke-free law campaign were positively related to web registrations. Each additional week that the cessation campaign ran on television resulted in an additional 34 web registrations. An additional seven people registered on the web for each additional week of the radio cessation campaign. Each additional week that the smoke-free law campaign was advertised on the radio resulted in 51 additional callers. Furthermore, there is a significant association between web registrations in 1 week and quitline call volume for the subsequent week (See Table 3).

Table 3 presents the standardized betas and partial eta squared values for question two. For quitline calls, we find that the previous week's call volume had the strongest relation to current week calls and the previous week's web registration levels were also strongly related. In terms of media predictors, the online cessation campaign, and print secondhand smoke campaigns were related to call volume. The New Year time period was marginally significantly related to weekly calls to the quitline.

A greater number of predictors were significantly related to web registration levels. The previous week's web registration levels were most highly related to the current week's web registration levels. The price of gas was also strongly related to web registration levels. Web program registration levels appear to be more highly influenced by media and events than were quitline call volumes. The television cessation campaign was the most strongly related media predictor. The radio campaigns for the smoke-free law and cessation were both significantly related. We also found that the New Year time period was strongly related to web program registrations, along with the Great American Smokeout, the QUITPLAN Services booth at the Minnesota State Fair, and the implementation of a 75 cent per pack price increase (Health Impact Fee).

We also found a significant relation between the secondhand smoke radio campaign and web registrations, although not in the anticipated direction. For each additional week that the secondhand smoke campaign ran on the radio the number of web registrations decreased by 29. The secondhand smoke radio campaign had a limited run and occurred around times when either other events or other media occurred. It appears that after controlling for the impact of those other factors, the periods during which the secondhand smoke radio ads were run tended to be lower web registration time periods; therefore, we find a spurious correlation between the radio ads being run and web registrations falling. We do not have any reason to suspect that running the ads actually caused people to decide not to enroll in the web program.

For question two, the multivariate regression results for each of the fitted models were statistically significant, *F*(17, 119) = 9.33, *p *< .001, adj. R-squared = 0.51 (Call Volume); and *F*(17, 119) = 22.29, *p *< .001, adj. R-squared = 0.73 (Web Registrations).

## Discussion

This study examined the relation between anti-tobacco media advertising exposure, calls to a stop-smoking quitline, and registrations in an online cessation program. We found a significant, positive relation between overall advertisement levels and quitline call volumes and web cessation program registrations. These findings related to weekly broadcast TRPs and call volume are consistent with prior research [12-15]. The models did find, however, a stronger relation between media and web registrations than with quitline calls. Further research is needed to understand how this finding may impact decisions for tailoring advertisement exposure when promoting multiple cessation offerings.

We also found an increase in quitline calls the week following increases in web registrations. It may be that tobacco users are exploring service options on the web and then contacting the quitline once they acquire the information they are seeking. This finding suggests that practitioners should carefully consider current Internet-use practices and how tobacco users utilize web-based cessation programs in creating cessation programs and media campaigns to promote them. It also points to the web as a valuable tool to promote and describe services as well as provide cessation intervention. Further research is needed to examine the evolving role of the Internet and how people use it to explore a product or service before making a commitment to fully engage in a program and/or interact with a live person.

Results of this study also suggest that communicating through a variety of types of media and with different messages to motivate tobacco users to seek services is important. Online and print advertising were positively related to quitline calls, while television and radio ads were positively related to web registrations. It may be that the target audience turns to the web to seek out the branded quitting service they see or hear promoted on television or radio while those accessing web and print advertising are calling the quitline as a result of a direct, visual cue for the quitline telephone number. Finally, developing mass media campaigns that offer a variety of messages including "why to quit" and "how to quit" are important to increasing service volume. The cessation and secondhand smoke campaigns increased calls to the quitline, and all three campaigns (cessation, secondhand smoke and smoke-free law) increased web registrations. Research is needed to further explore what combinations of media types and messages ensures the broadest reach with tobacco users who are diverse in media consumption patterns and motivations to quit.

Beyond the types of campaigns and their placements, economic stress and cessation-related events may have a large impact on seeking assistance to quit smoking. In both of the models, the price of gas, Great American Smokeout, and New Year time period were all related to calls, web registrations or both. Given the potential to plan media promotions in ways that leverage these external events, further analyses should be conducted to better understand the impacts of economic stress and other cessation-related events.

### Limitations

There are several limitations to consider. First, TRPs were available for television and radio advertisements; however, TRPs are not used to measure other types of media. Therefore, we coded non-broadcast media as on and off for each week during the study period, and when including both broadcast and non-broadcast media in the second analysis, we transformed broadcast TRPs into weekly indicator variables. This puts all the measures on the same unit of analysis, however it may limit our ability to fully assess the impact of these factors. Similarly, earned media events were measured with binary on-off codes, again limiting the explanatory power of the events.

Second, the models in this study did not include data indicating when free nicotine replacement therapy was being advertised due to a lack of complete data on NRT tagging for the ads included in the models. Regardless, we believe free NRT is an attractive offer for smokers and may be related to people calling the quitline beyond the impact of other factors. This analysis would be strengthened if these data were included.

Finally, while we included information about the number of callers and web registrants associated with increased levels of TRPs for the purpose of illustrating the relative impact of various media on service volumes, these findings are specific to this particular context and do not account for the multitude of other factors that need to be considered for media planning purposes. In particular, the quality of the creative and the specific messages of particular ads are not examined in these analyses.

## Conclusions

Developing mass media campaigns that offer a variety of messages and communicating through different types of media to motivate tobacco users to seek services appears important to reach tobacco users--a diverse group with multiple motivation triggers for seeking services. Media types and campaigns impacted service volume for the quitline and web differently, suggesting a need for further research to better understand the complexities and opportunities involved in promoting cessation programs that offer both telephone and web-based cessation services.

## Competing interests

This study was funded by ClearWay Minnesota.

## Authors' contributions

BS, AM, ML & MC designed this study; AZ, & MC conducted the data analysis; BS, AM, LG, ML and MC drafted sections of the initial manuscript; all authors participated in the interpretation of the results and were involved in revising the manuscript; all authors read and approved the final manuscript.

## Pre-publication history

The pre-publication history for this paper can be accessed here:

http://www.biomedcentral.com/1471-2458/11/939/prepub

## Supplementary Material

Additional file 1 **Supplementary figures**. Figure S1. Paid media, earned media, and service volumes included in statistical models, July 2005 - June 2006. Figure S2. Paid media, earned media, and service volume included in statistical models, July 2006 - June 2007. Figure S3. Paid media, earned media, and service volumes included in statistical models, July 2007 - June 2008. Click here for file
