# Supplementary material for: The relation between media promotions and service volume for a statewide tobacco quitline and a web-based cessation program
Source: BMC Public Health. 2011 Dec 16;11:939. doi: 10.1186/1471-2458-11-939 (PMC3299703; doi:10.1186/1471-2458-11-939)

Figure S1. Paid media, earned media, and service volumes included in statistical models, July 2005 - June 2006

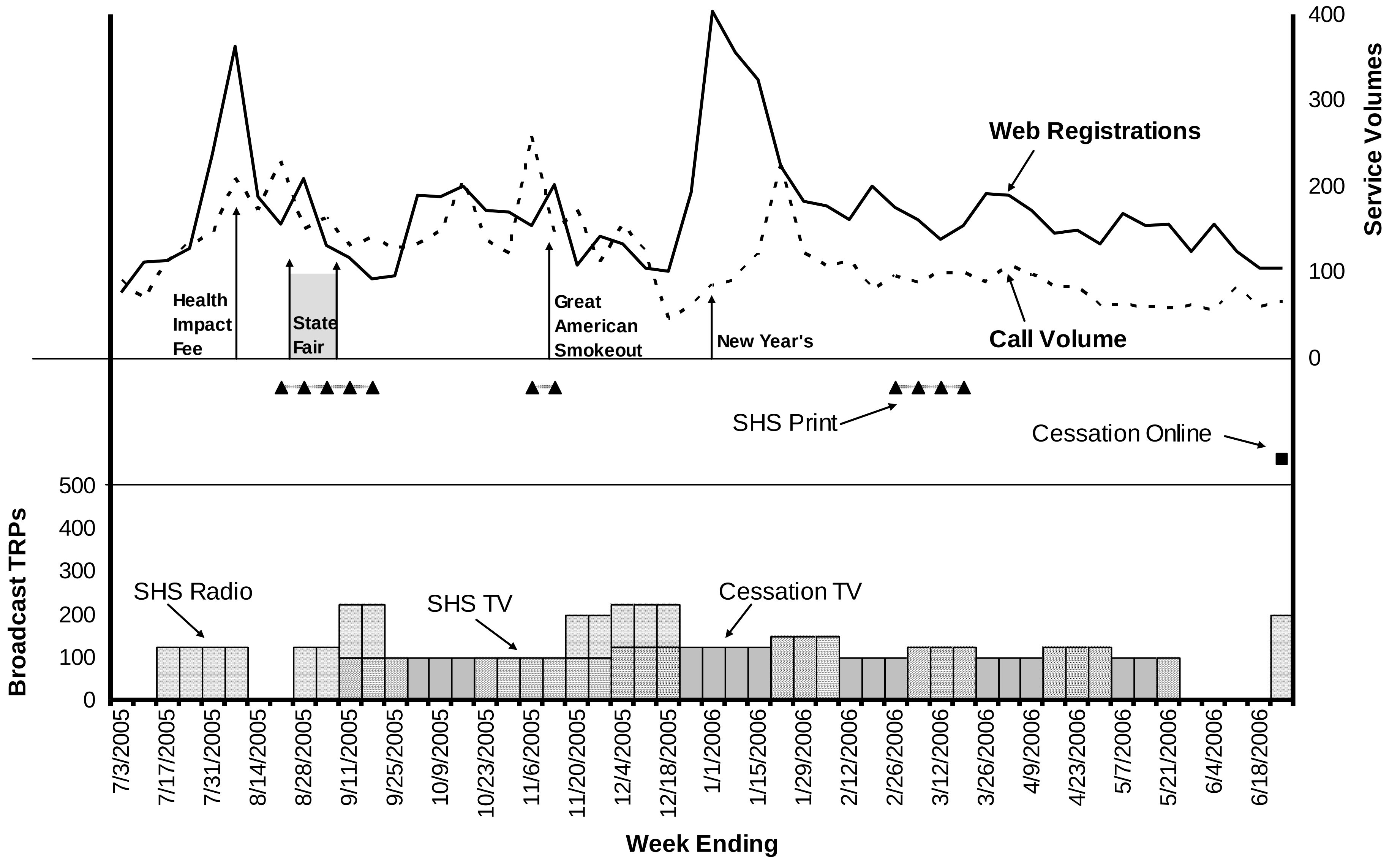

Figure S2. Paid media, earned media, and service volume included in statistical models, July 2006 - June 2007

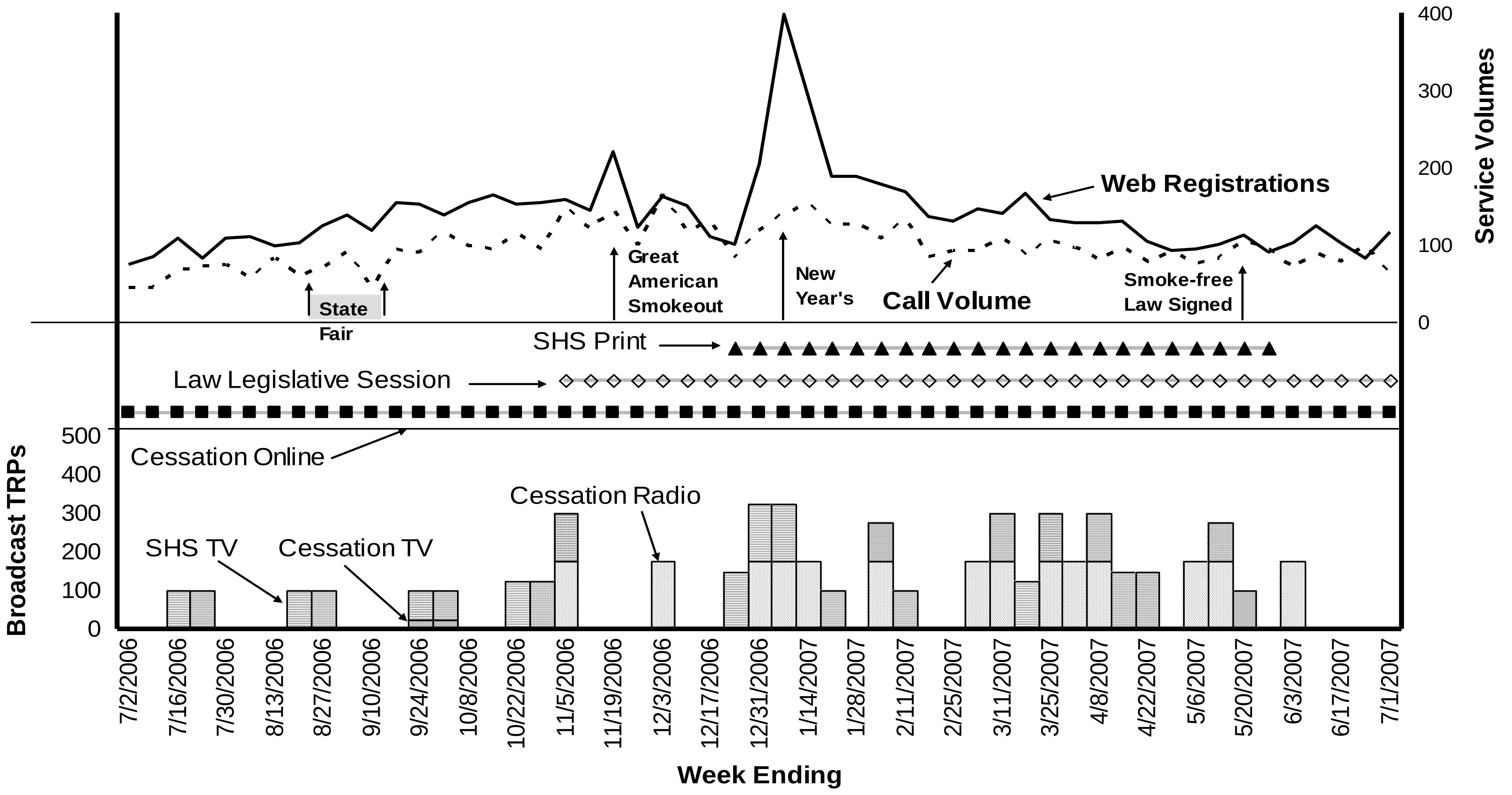

Figure S3. Paid media, earned media, and service volumes included in statistical models, July 2007 - June 2008

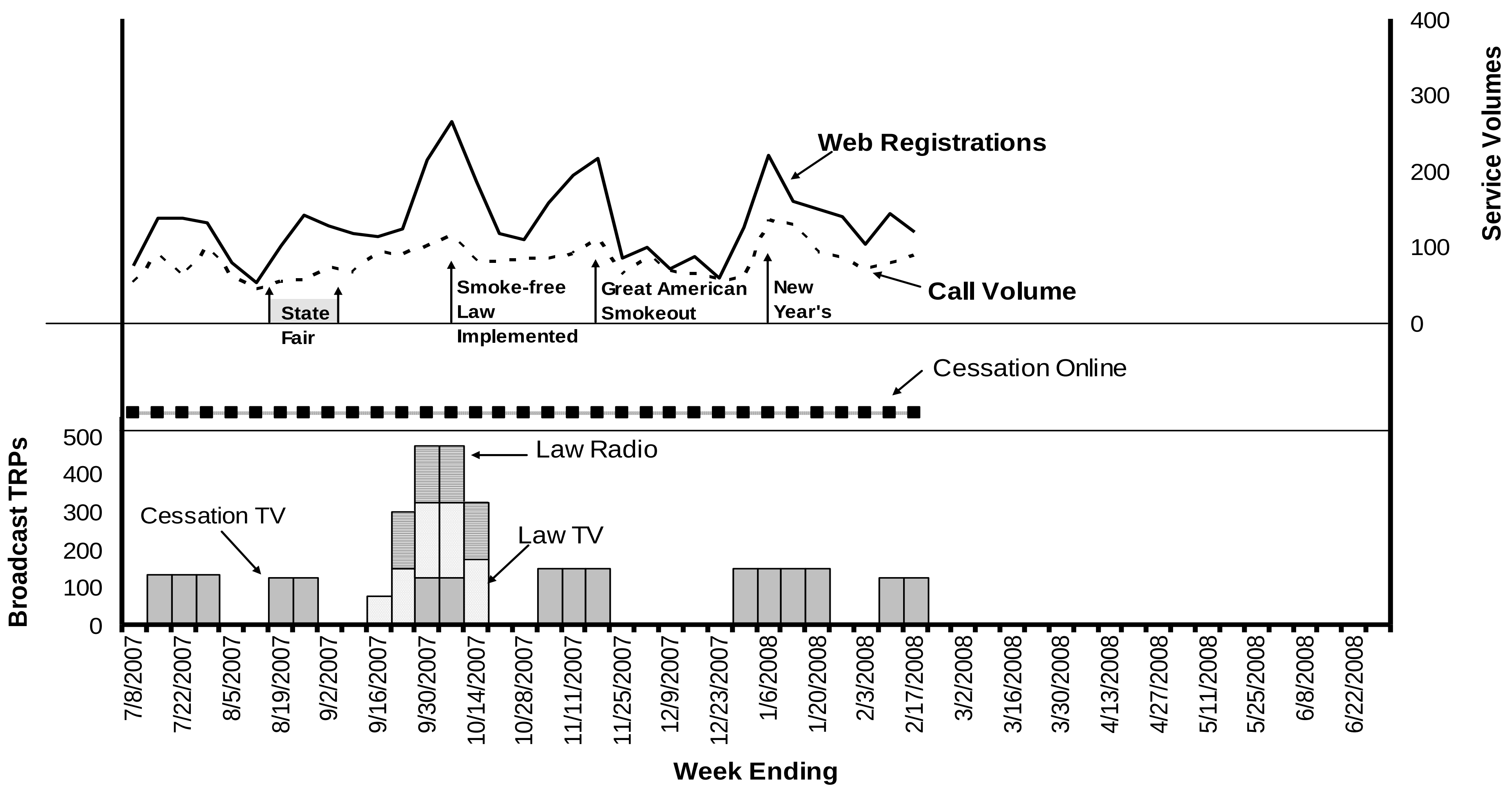

Supplement: Additional file 1 — Supplementary figures. Figure S1. Paid media, earned media, and service volumes included in statistical models, July 2005 - June 2006. Figure S2. Paid media, earned media, and service volume included in statistical models, July 2006 - June 2007. Figure S3. Paid media, earned media, and service volumes included in statistical models, July 2007 - June 2008. [file 1471-2458-11-939-S1.PDF]
